# High-density lipoprotein protects cardiomyocytes against necrosis induced by oxygen and glucose deprivation through SR-B1, PI3K, and AKT1 and 2

**DOI:** 10.1042/BCJ20170703

**Published:** 2018-04-05

**Authors:** Kristina K. Durham, Kevin M. Chathely, Bernardo L. Trigatti

**Affiliations:** 1 Thrombosis and Atherosclerosis Research Institute, McMaster University and Hamilton Health Sciences, 237 Barton St. E. Hamilton, Ontario, Canada L8L 2X2; 2 Department of Biochemistry and Biomedical Sciences, McMaster University, 1280 Main St W. Hamilton, Ontario, Canada L8S 4L8

**Keywords:** cardiomyocytes, cell death, high-density lipoprotein

## Abstract

The cardioprotective lipoprotein HDL (high-density lipoprotein) prevents myocardial infarction and cardiomyocyte death due to ischemia/reperfusion injury. The scavenger receptor class B, type 1 (SR-B1) is a high-affinity HDL receptor and has been shown to mediate HDL-dependent lipid transport as well as signaling in a variety of different cell types. The contribution of SR-B1 in cardiomyocytes to the protective effects of HDL on cardiomyocyte survival following ischemia has not yet been studied. Here, we use a model of simulated ischemia (oxygen and glucose deprivation, OGD) to assess the mechanistic involvement of SR-B1, PI3K (phosphatidylinositol-3-kinase), and AKT in HDL-mediated protection of cardiomyocytes from cell death. Neonatal mouse cardiomyocytes and immortalized human ventricular cardiomyocytes, subjected to OGD for 4 h, underwent substantial cell death due to necrosis but not necroptosis or apoptosis. Pretreatment of cells with HDL, but not low-density lipoprotein, protected them against OGD-induced necrosis. HDL-mediated protection was lost in cardiomyocytes from SR-B1^−/−^ mice or when SR-B1 was knocked down in human immortalized ventricular cardiomyocytes. HDL treatment induced the phosphorylation of AKT in cardiomyocytes in an SR-B1-dependent manner. Finally, chemical inhibition of PI3K or AKT or silencing of either AKT1 or AKT2 gene expression abolished HDL-mediated protection against OGD-induced necrosis of cardiomyocytes. These results are the first to identify a role of SR-B1 in mediating the protective effects of HDL against necrosis in cardiomyocytes, and to identify AKT activation downstream of SR-B1 in cardiomyocytes.

## Introduction

Cardiovascular disease is a leading cause of death and a major burden to the healthcare systems of developed societies [1]. About half of all cardiovascular disease-related deaths are due to coronary heart disease [1]. Partial or complete occlusion in one or more areas of the coronary arteries renders an ischemic myocardial environment, which, if left unperfused, results in infarction due to irreversible cardiomyocyte death and myocardial infarction. Over time, the increased demand on the non-infarcted tissue may lead to detrimental functional changes and heart failure; therefore, it is of utmost importance to prevent or limit the amount of infarcted tissue.

Low plasma HDL (high-density lipoprotein) cholesterol is associated with increased risk of mortality in patients recovering from myocardial infarction [2], and HDL cholesterol levels are inversely correlated with cardiovascular disease risk [3]. The clinical data are supported by *ex vivo* experimental evidence, where administration of HDL provides protection against ischemia–reperfusion injury in rodent hearts [4,5], and *in vivo* experimental evidence demonstrates that overexpression of apolipoprotein (apo) A1 (the major protein component of HDL) protects low-density lipoprotein (LDL) receptor (LDLR^−/−^) knockout mice from coronary artery ligation-induced myocardial infarction, cardiac dysfunction, and death [6]. Conversely, apoA1-deficiency, and a resultant reduction in HDL, impairs cardiomyocyte mitochondrial function and results in larger infarctions following coronary ligation in mice [7].

Although the physiological effects of HDL against myocardial ischemia are well documented in both humans and rodents, the receptor(s) and pathways through which HDL induces protection at the cardiomyocyte are not completely defined. The scavenger receptor class B type 1 (SR-B1) is a high-affinity HDL receptor that is present in heart tissue, and has been implicated in mediating HDL-dependent cytoprotective intracellular signaling, including activation of the PI3K (phosphatidylinositol-3-kinase)/AKT signaling pathway, in diverse cell types including endothelial cells, macrophages, and Chinese hamster ovary cells [8–14]. PI3K and AKT are well-characterized mediators of cardiomyocyte survival [15–18]. The requirement for SR-B1 in HDL-mediated induction of PI3K/AKT signaling in cardiomyocytes or its role in protection of cardiomyocytes against ischemic injury has not been tested.

Coronary artery atherosclerosis and non-lethal myocardial infarcts can be induced in LDLR/Apo E double knockout (dKO) mice by feeding them a high fat diet for extended periods of time [19]. On the other hand, coronary artery atherosclerosis and lethal myocardial infarcts develop spontaneously in mice lacking SR-B1 and ApoE (SR-B1/ApoE dKO) [20] and in a high fat/high cholesterol diet-dependent manner in SR-B1/LDLR dKO mice [21] or SR-B1^−/−^ mice with a hypomorphic mutation in apoE [22]. In SR-B1-deficient models, spontaneous or diet-induced coronary artery atherosclerosis and myocardial infarction are accompanied by cardiac conductance abnormalities, reduced heart function, and early death [20–22]. The striking phenotype of extensive myocardial infarction when SR-B1 is knocked out in atherosclerosis-susceptible strains of mice led us to hypothesize that the effects of SR-B1 extend beyond its role in atherosclerosis, and that SR-B1 may play a more direct role in protecting cardiomyocytes against myocardial infarction.

In the present study, we directly tested the role of SR-B1 in HDL-activated PI3K/AKT signaling in cardiomyocytes and protection against oxygen and glucose deprivation (OGD)-induced cardiomyocyte death.

## Materials and methods

### Mice

All procedures involving animals were approved by the McMaster University Animal Research Ethics Board and were in accordance with guidelines from the Canadian Council on Animal Care. Mice were bred and housed in ventilated cages with automatic watering and free access to food in the Division of Comparative Medicine Facility of the Thrombosis and Atherosclerosis Research Institute. SR-B1^−/−^ mice (backcrossed >10 generations on C57BL/6 genetic background) were bred as homozygous mutants and breeders were fed a diet of chow containing 0.5% probucol [23]. Founders were originally obtained from Monty Krieger (Massachusetts Institute of Technology, Cambridge, MA, U.S.A.). Wild-type C57BL/6 (SR-B1^+/+^) mice were bred from founders originally obtained from the Jackson Laboratories (Barr Harbor, ME, U.S.A.).

### Culture of neonatal mouse cardiomyocytes and human immortalized ventricular cardiomyocytes

Neonatal mouse cardiomyocytes (NMCMs) were isolated from hearts of 1- to 3-day-old mice. Briefly, neonatal mice were killed by decapitation and hearts were excised and washed with Hank's balanced saline solution (HBSS, Life Technologies, Burlington, ON, Canada). The ventricles were serially digested at 37°C using 0.2 mg/ml collagenase type II (Worthington Biochemical Corp., Lakewood, NJ, U.S.A.) and 0.6 mg/ml pancreatin (Sigma–Aldrich Canada Co., Oakville, ON, Canada) in HBSS. The cell suspension was centrifuged at 300×***g*** for 10 min at 4°C, the supernatant was removed, and the cell pellet was resuspended in 37°C DMEM (Dulbecco's modified Eagle's medium)/M199 (4 : 1) medium supplemented with 10% horse serum, 5% fetal bovine serum (FBS), 100 µM bromo-deoxyuridine, 100 U/ml penicillin, 100 µg/ml streptomycin, and 0.5 mM l-glutamine (Life Technologies, Burlington, ON, Canada). Non-cardiomyocytes were removed by plating the cell suspension on an uncoated plate for 1 h. The myocyte-enriched suspension was collected and then plated at 1 × 10^5^_ _cells/cm^2^ on collagen type I (Life Technologies, Burlington, ON, Canada)-coated dishes and incubated in a humid 5% CO_2_ incubator at 37°C for 48 h at which point they were used for experiments. SV40 large T-antigen immortalized human ventricular CMs (HIVCMs) were obtained from Applied Biological Materials, Inc. (Richmond, BC, Canada). HIVCMs were cultured in Prigrow I media (Applied Biological Materials, Inc., Richmond, BC, Canada) supplemented with 10% FBS and 100 U/ml penicillin.

### Oxygen and glucose deprivation

Forty-eight hours after plating, cell culture media were replaced with media containing 3% newborn calf lipoprotein-deficient serum, and cells were cultured for a further 24 h; all experiments were carried out in media supplemented with lipoprotein-depleted serum [14]. Cells were incubated with or without 100 µg (protein)/ml human HDL or LDL (Alfa Aesar, Ward Hill, MA, U.S.A.) for 30 min, then media were changed to OGD media (DMEM with no glucose and 1% lipoprotein-deficient serum), and cells were incubated in a humidified chamber with 100% N_2_ at 37°C for 4 h. Alternatively, normoxic control cells received DMEM containing glucose and 1% lipoprotein-deficient serum, and were incubated in 95% air, 5% CO_2_ at 37°C. In some experiments, HIVCMs were transfected with 30 nM siRNA in the presence of Lipofectamine RNAi Max (Life Technologies, Burlington, ON, Canada), 24 h prior to lipoprotein starvation. SiRNA targeting human SR-B1 (siSR-B1: Hs_SCARB1_6), AKT1 (siAKT1: Hs_AKT1_10) and AKT2 (siAKT2: Hs_AKT2_7), and AllStars Negative Control siRNA (siCntrl) were purchased from Qiagen, Inc. (Toronto, ON, Canada). In other experiments, cells were treated with 10 µM LY294002 (PI3K inhibitor; Cell Signaling Technologies, Inc., Danvers, MA), 3 µM AKT inhibitor V (pan-AKT inhibitor; Millipore Canada Ltd, Etobicoke, ON, Canada), 10 µM Nec-1s (necroptosis inhibitor; BioVision, Inc., Milpitas, CA), or vehicle (DMSO, Sigma–Aldrich Canada Co., Oakville, ON, Canada). All inhibitors were applied to cells 10 min prior to other treatments (including HDL) and for the duration of OGD treatment.

### Measurement of cell viability

Cells were cultured and treated in a 96-well plate. Following OGD, cells were incubated with the Cell Titer Blue (Promega Corp., Madison, WI, U.S.A.) assay reagent according to the manufacturer's instructions to assess cell viability using a spectrophotometer. Cell viability was expressed as a percentage of the viability of parallel cultures of cells not exposed to OGD.

### Measurement of necrosis

Following OGD, cells were stained with propidium iodide (PI), washed extensively, fixed with 4% paraformaldehyde, and counterstained with 4′,6-diamidine-2′-phenylindole dihydrochloride (DAPI) to identify nuclei. Necrotic cells were identified as having PI-stained nuclei. Additionally, NMCMs were co-stained with a mouse monoclonal anti-cardiac troponin T antibody (cTnT, MA5-12960, Fisher Scientific, Inc., Waltham, MA, U.S.A.) using a Mouse on Mouse Kit (Vector Laboratories, Burlington, ON, Canada), biotinylated secondary, and tertiary labeling with streptavidin conjugated with AlexaFluor 488 (Life Technologies, Burlington, ON, Canada) to identify cardiomyocytes.

### Measurement of apoptosis

Following treatment, apoptosis was detected by staining fixed cells by terminal deoxynucleotidyl transferase nick end labeling (TUNEL) using the ApopTag Plus Fluorescein In Situ Apoptosis Detection Kit (Millipore (Canada) Ltd, Etobicoke, ON, Canada) or by staining unfixed cells with fluorescein isothiocyanate (FITC)-conjugated Annexin V (AxV, Life Technologies, Burlington, ON, Canada) and PI according to the manufacturer's instructions. All cells were then fixed and counterstained with DAPI as above.

### Microscopy and quantification of necrotic and apoptotic cells

Fluorescent images were captured using a Zeiss Axiovert 200M inverted fluorescence microscope (Carl Zeiss Canada Ltd, Toronto, ON, Canada) or an Olympus BX41TF fluorescence microscope (Olympus Canada, Inc., Richmond Hill, ON, Canada). A minimum of 4 and maximum of 10 fields of view were imaged per well. Necrotic nuclei were identified as having PI nuclear staining, and apoptotic cells were identified as having AxV staining. Necrotic and apoptotic cell numbers were counted across 4–10 fields of view for each well and were averaged across triplicate wells. Percentage ratios of the numbers of PI/total (PI + DAPI)-stained nuclei were taken as measures of the proportions of necrotic cells and percentage ratios of the numbers of AxV-stained cells/DAPI-stained nuclei or TUNEL-stained/DAPI-stained nuclei were taken as measures of the proportions of apoptotic cells.

### Immunoblotting

Cells were lysed in a buffer containing 20 mM Tris–HCl (pH 7.5), 150 mM NaCl, 1 mM Na_2_EDTA, 1 mM EGTA, 1% Triton, 2.5 mM Na_4_P_2_O_7_, 1 mM β-glycerophosphate, 1 mM Na_3_VO_3_, with 1 µg/ml pepstatin A, 1 mg/ml leupeptin 2 µg/ml aprotinin and 50 µM *p*-amidinophenylmethylsulfonylfluoride (all from Sigma–Aldrich Canada Co., Oakville, ON, Canada). Samples (20–25 µg of total protein) prepared from lysates were subjected to SDS–polyacrylamide gel electrophoresis, transferred onto a PVDF membrane, and analyzed by immunoblotting as previously described [14]. The antibodies used (and catalog numbers) were: rabbit antibodies for horseradish peroxidase (HRP)-conjugated β-actin (12620), total AKT (9272), AKT1 (2938), AKT2 (3063), and phosphorylated AKT at Ser 473 (4060) (Cell Signaling Technology, Danvers, MA, U.S.A.); rabbit anti-SR-B1 (NB400-104; Novus Biologicals, Oakville, ON, Canada); and HRP-conjugated donkey anti-rabbit IgG (711-035-152; Jackson ImmunoResearch, West Grove, PA, U.S.A.). HRP was detected by enhanced chemiluminescence (GE Healthcare Life Sciences, Baie d'Urfe, QC, Canada) and quantified using a Gel Doc instrument (Bio-Rad Laboratories, Hercules, CA, U.S.A.). For analysis of AKT phosphorylation, blots were first probed with the anti-phospho-AKT primary and secondary HRP-conjugated anti-rabbit antibody, stripped, and then reprobed with the total AKT primary and secondary HRP-conjugated anti-rabbit antibody. All blots were stripped and reprobed using the HRP-conjugated anti-β-actin antibody.

### Statistics

All data are expressed as means ± SEM. Data were graphed and statistically analyzed using GraphPad Prism (GraphPad Software, Inc., La Jolla, CA). Data comparing two groups were tested for significance by Student's *t*-test, and data comparing multiple groups were tested using one-way ANOVA and Tukey's multiple comparisons test. A value of *P* < 0.05 was considered statistically significant.

## Results

### HDL protects against OGD-induced necrosis

**Figure 1.**
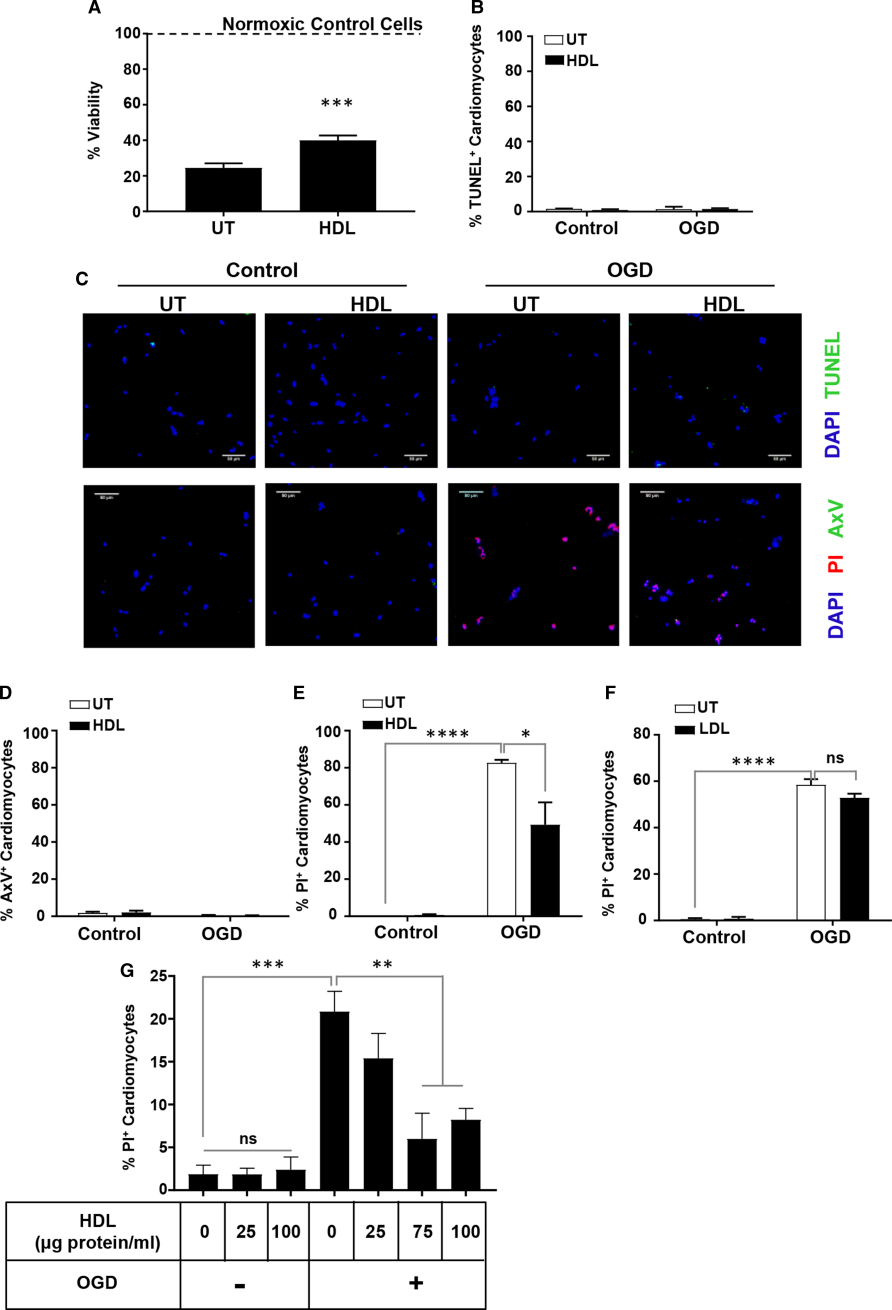
HDL protects HIVCMs against OGD-induced necrosis. (**A**) Cell viability measured by the Cell Titer Blue viability assay for HIVCM treated for 30 min without or with human HDL (100 μg protein/ml) and then exposed to OGD for 4 h. The dashed line indicates 100 % viability, determined by the measurement of cell viability in control cells not exposed to OGD. *n* = 10 wells/group. ***P < 0.001. (**B**) Quantification and (**C**, top row) representative images of TUNEL- (green) and DAPI- (blue) stained HIVCM. *n* = 3 wells/group. (**C**, bottom row) Representative images of FITC-AxV- (green), PI- (red), and DAPI- (blue) stained HIVCM treated with or without HDL (30 min) and exposed to 4 h of OGD or control normoxic conditions (as indicated). All scale bars = 50 µm. Quantification of OGD-induced cell death by FITC-AxV staining (**D**) or PI staining (**E**) in HIVCM treated as above. (**F**) Quantification of OGD-induced cell death by PI staining in HIVCM pretreated for 30 min with human LDL (100 μg protein/ml) and then exposed to OGD for 4 h. *n* = 3 wells/group. (**G**) HIVCM cells were pretreated with the indicated concentrations of human HDL and then either maintained under normoxic conditions (OGD−) or exposed to OGD for 4 h (OGD+) and stained with PI to detect necrosis. *n* = 3 wells/group. Data represents means ± SEM. ****P < 0.0001, ***P < 0.001, **P < 0.01, *P < 0.05, ns, not statistically significant (*P* > 0.14). Statistical analysis was carried out by unpaired Student's *t*-test (**A**) or one-way ANOVA (**B**, **D–G**).

Exposure of HIVCM to 4 h of OGD resulted in a dramatic reduction in their viability, to 25 ± 2.6% compared with untreated (‘normoxic’) cells (Figure 1A). Pretreatment of cells with HDL for 30 min, prior to exposure to OGD, however, resulted in an attenuated reduction in viability after 4 h of OGD, to 40 ± 2.7% compared with untreated cells (Figure 1A). Cells subjected to OGD exhibited only very low levels of apoptosis, as measured by TUNEL or AxV staining, and these very low levels were unaffected by HDL treatment (Figure 1B–D). The lack of cardiomyocyte apoptosis following 4 h of OGD in our experiment is consistent with a recent report that 4 h of OGD induced significant cell death with little detectable apoptosis in neonatal rat ventricular cardiomyocytes [24]. Despite the low levels of apoptosis, exposure of HIVCM to 4 h of OGD caused membrane permeabilization (a marker of necrotic or necroptotic cell death) as evidenced by increased nuclear staining with PI compared with corresponding normoxic controls (82.6 ± 1.7 vs. 0 ± 0% PI-positive cardiomyocytes, Figure 1E,G), and treatment with HDL attenuated PI uptake (49.2 ± 12.1 vs. 82.6 ± 1.7% PI-positive cardiomyocytes, Figure 1E,G). Conversely, in a separate experiment, treatment with LDL did not protect HIVCM against OGD-induced cell death as measured by PI staining (58.4 ± 2.5 vs. 52.8 ± 1.8% PI-positive cardiomyocytes, Figure 1F). Treatment of cardiomyocytes with increasing concentrations of HDL prior to OGD exposure revealed that HDL-dependent protection against OGD-induced necrosis exhibited HDL concentration dependence and appeared to saturate by 75 and 100 µg HDL (protein)/ml (Figure 1G).

**Figure 2.**
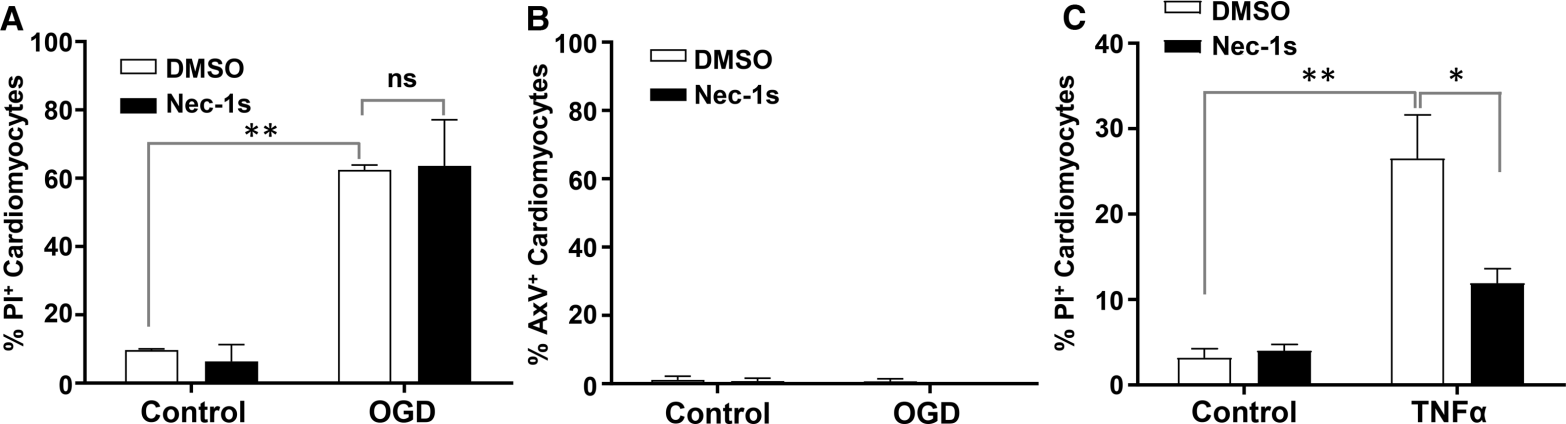
Nec-1s, an inhibitor of necroptosis, does not prevent OGD-induced death of HIVCM. HIVCM cells were treated with or without 10 μM Nec-1s, an inhibitor of necroptosis, 10 min prior to and during exposure to OGD for 4 h (**A** and **B**) or (**C**) 24 h treatment with TNF-α (100 ng/ml). Control cells were treated with an equivalent volume of DMSO solvent (final concentration of 0.1%). (**A**) Quantification of the proportion of PI-positive cells (**A**) or AxV-positive cells (**B**) after 4 h of OGD. (**C**) Quantification of the proportion of PI-positive cells after 24 h of exposure to TNF-α. Data represent means ± SEM of *n* = 3 replicates. **P < 0.004; *P = 0.02; ns, not statistically significant (*P* > 0.99), analyzed by one-way ANOVA.

Nuclear accumulation of PI stain could represent cell death due to either necrosis or necroptosis (a form of programmed necrotic cell death), as membrane permeabilization is a feature of both [25]. To distinguish between these two types of cell death, cells were treated with the necroptosis inhibitor Nec-1s. Nec-1s did not reduce PI uptake following OGD (62.5 ± 1.4 vs. 63.6 ± 13.6% PI-positive cardiomyocytes, Figure 2A). As in Figure 1, little to no AxV staining was detected following OGD, indicating minimal contribution of apoptosis (Figure 2B). Nec-1s treatment did, however, substantially reduce PI uptake following exposure to TNF-α (Figure 2C), a known inducer of necroptosis [25]. This suggested that the PI uptake following OGD is a consequence of unprogrammed, necrotic cell death, and not necroptosis.

### HDL requires SR-B1 to protect against OGD-induced cell death

**Figure 3.**
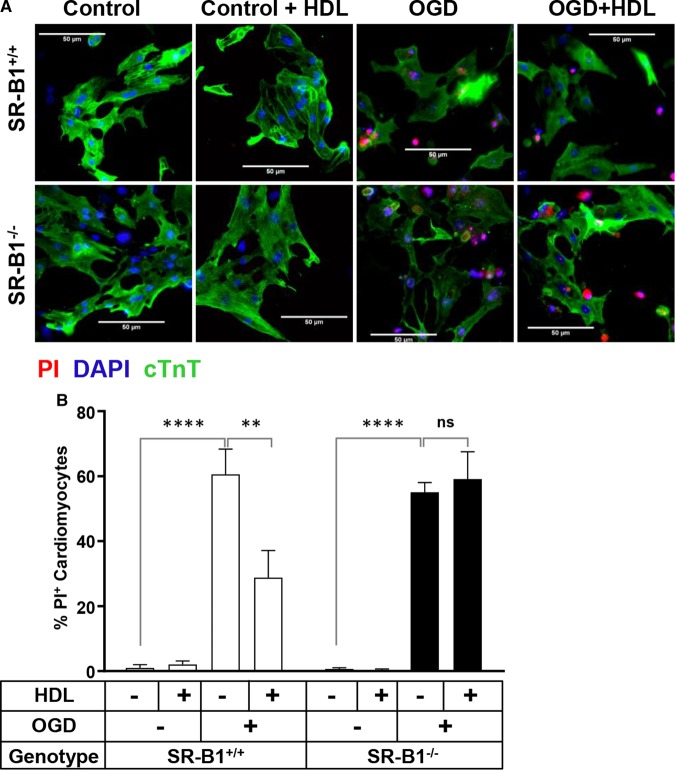
SR-B1 is required for HDL-mediated protection of NMCM against OGD-induced necrosis. (**A**) Representative images and (**B**) quantification of PI staining in SR-B1^+/+^ and SR-B1^−/−^ NMCM treated for 30 min with or without HDL (100 μg/ml) prior to exposure to OGD for 4 h. Control cells were maintained in normal media under normoxic conditions. In (**A**), cells were stained prior to fixation with PI (red) or, after fixation, immunostained for cTnT (green) and stained with DAPI (blue). Scale bars = 50 μm. (**B**) Data represent means ± SEM for *n* = 3 replicates. ****P < 0.0001; **P < 0.01; ns, not statistically significant (*P* > 0.99) by one-way ANOVA.

**Figure 4.**
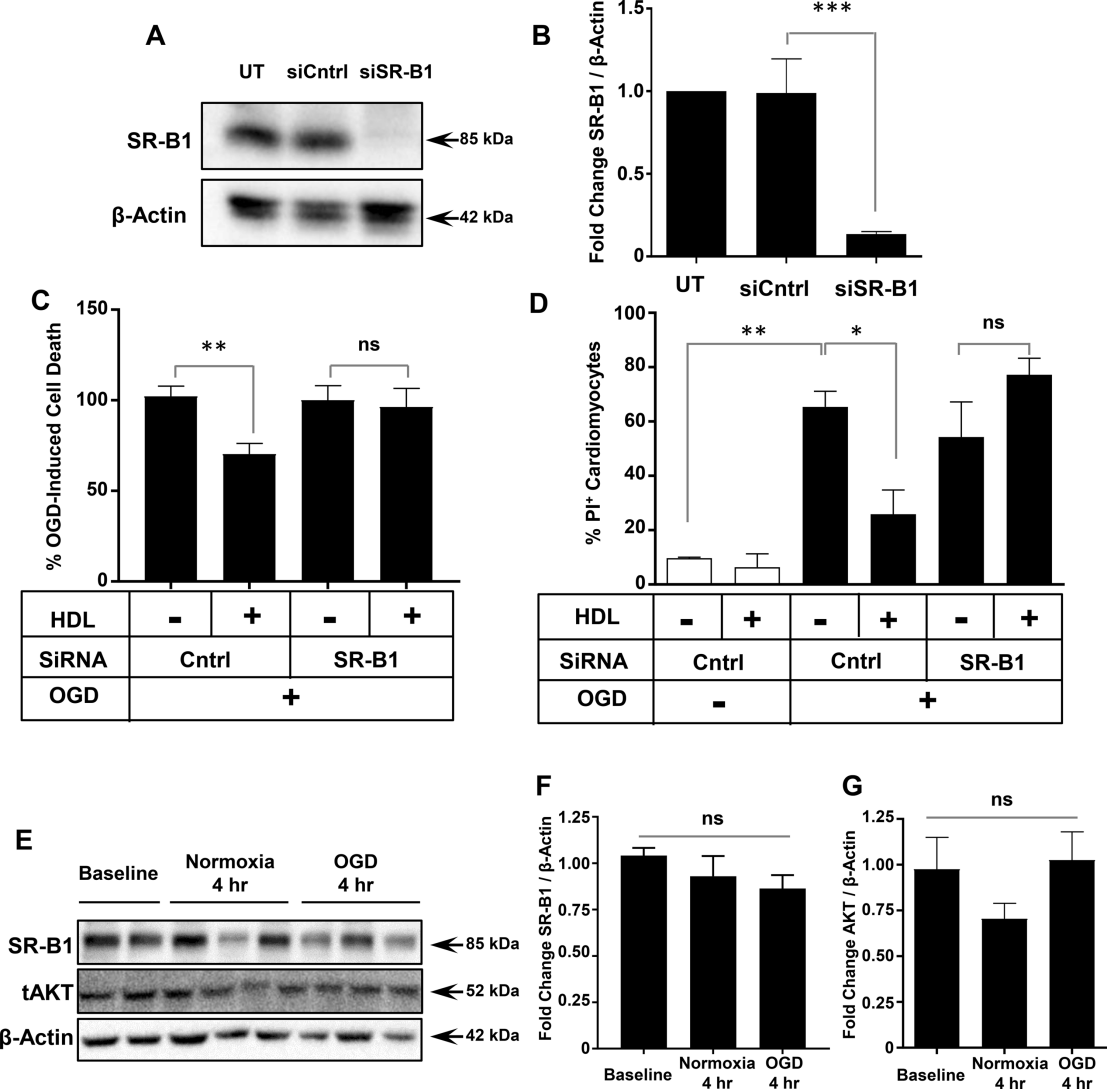
siRNA-mediated knock down of SR-B1 impairs HDL-mediated protection of HIVCM against OGD-induced necrosis. (**A**) Representative immunoblot and (**B**) quantification (*n* = 3) of SR-B1 knockdown by siRNA in HIVCM. UT: untransfected cells; siCntrl: transfected with control siRNA; siSR-B1: transfected with siRNA targeting SR-B1. (**C**) Quantification of the percentage of OGD-induced cell death in siCntrl- or siSR-B1-transfected HIVCMs treated with or without HDL (100 μg/ml) prior to 4 h of OGD. Cell viability was measured using the Cell Titer Blue assay and the reduction in viability in siCntrl-transfected cells treated without HDL was set as 100% OGD-induced cell death (*n* = 9). (**D**) Proportion of PI-stained cells when HIVCMs were transfected with siCntrl or siSR-B1, treated for 30 min with or without HDL, and exposed to OGD for 4 h as above (*n* = 3). (**E**) Representative immunoblots of SR-B1, AKT, and β-actin and quantification of (**F**) SR-B1/β-actin (*n* = 5, 6, 6) and (**G**) AKT/β-actin (*n* = 3), expressed as fold change in arbitrary relative units. ***P = 0.0003; **P < 0.005; *P < 0.03; ns, not statistically significant (*P* > 0.3) by one-way ANOVA.

NMCMs were isolated from SR-B1^+/+^ and SR-B1^−/−^ mice to test the role of SR-B1 in facilitating HDL-mediated protection against OGD-induced necrosis. OGD led to similar levels of necrosis in SR-B1^+/+^ and SR-B1^−/−^ NMCMs (60.6 ± 7.7 vs. 55.1 ± 2.1% PI-positive cardiomyocytes, Figure 3). HDL pretreatment reduced the extent of OGD-induced necrosis by half in SR-B1^+/+^ NMCMs (28.5 ± 8.3% PI-positive cardiomyocytes, Figure 3), but failed to protect SR-B1^−/−^ NMCMs against OGD-induced necrosis (59.1 ± 8.4% PI-positive cardiomyocytes, Figure 3). This suggests that SR-B1 in murine cardiomyocytes is required for HDL-mediated protection against OGD-induced necrosis. To test if the same was true in HIVCMs, we used siRNA to knock down levels of SR-B1 protein by ∼87% (Figure 4A,B). This prevented HDL-dependent protection of HIVCM against OGD-induced cell death as measured by Cell Titer Blue viability staining (Figure 4C) or PI staining (Figure 4D). Exposure of HIVCM cells to OGD itself, however, did not alter the levels of SR-B1 as detected by immunoblotting (Figure 4E,F). These data demonstrate that cardiomyocyte SR-B1 is required for HDL-mediated protection against OGD-induced necrosis.

### HDL activates AKT in an SR-B1-dependent manner

**Figure 5.**
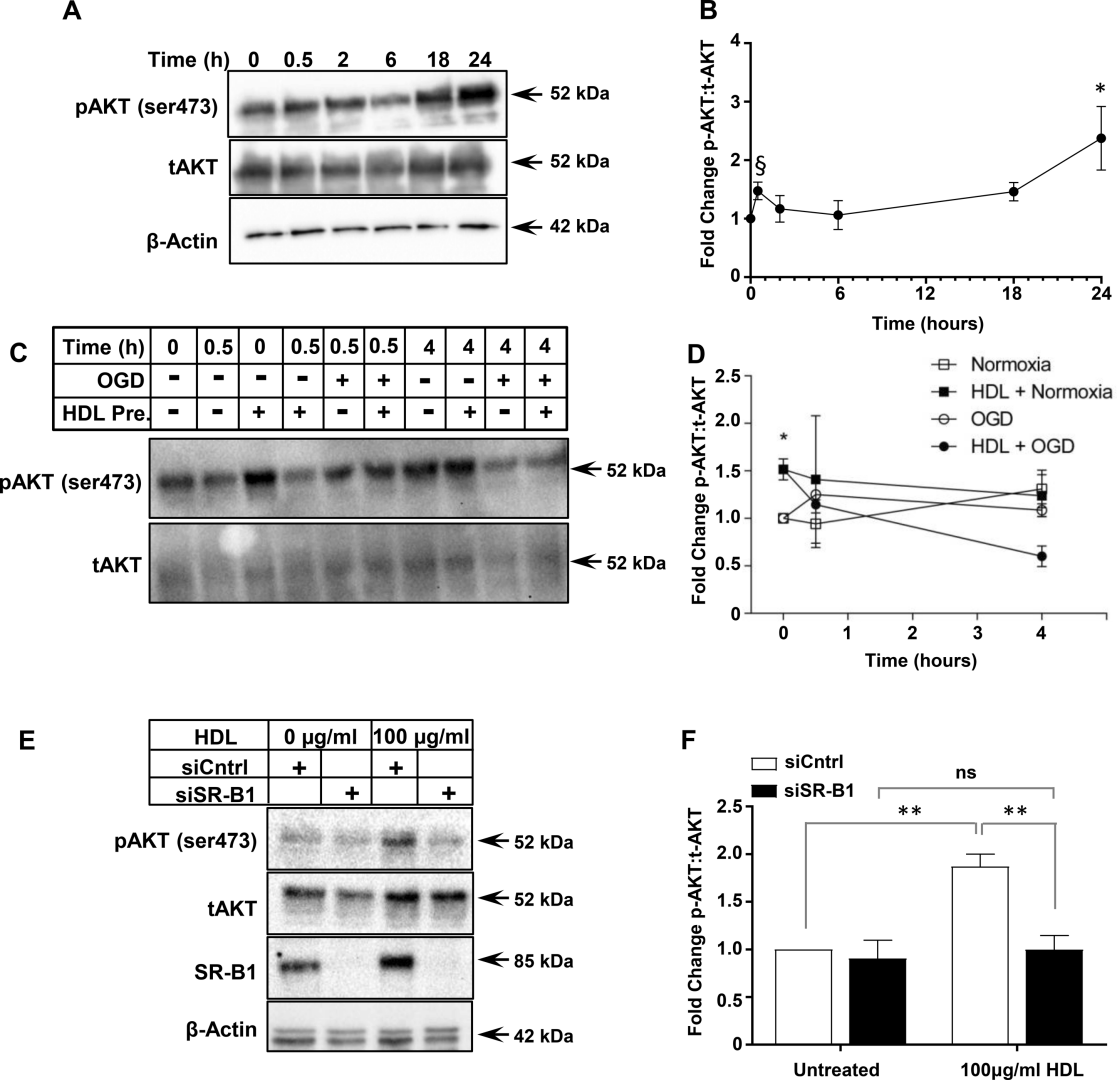
siRNA-mediated knock down of SR-B1 impairs HDL-stimulated AKT phosphorylation in HIVCMs. (**A**) Representative immunoblots of phospho-AKT (pAKT), total AKT (tAKT), and β-actin (loading control) and (**B**) quantification of the ratio of pAKT/tAKT (*n* = 3) in HIVCMs incubated with HDL (100 μg protein/ml) for different times. (**C**) Representative immunoblots of pAKT and tAKT and (**D**) quantification of the ratio of pAKT/tAKT (*n* = 3) in HIVCMs incubated with HDL (100 μg protein/ml) for 30 min prior to and during exposure to OGD or maintenance under normal conditions (‘Normoxia’). (**E**) Representative immunoblots of pAKT, tAKT, SR-B1, and β-actin (loading control) in siCntrl and siSR-B1-transfected HIVCMs incubated with or without HDL for 30 min. (**F**) Quantification of the ratio of pAKT/tAKT (expressed as fold change relative to untreated; *n* = 4). Data are means ± SEM. **P < 0.004; *P < 0.05; ns, not statistically significant (*P* > 0.9) by one-way ANOVA. ^§^*P* < 0.02 by unpaired Student's *t*-test, relative to 0 h time point.

Exposure of HIVCM cells to OGD for 4 h did not alter the levels of total AKT protein (Figure 4E,G). To examine the signaling pathways involved in SR-B1-mediated and HDL-dependent protection against OGD-induced necrosis, we incubated HIVCM with HDL and examined the extent of AKT phosphorylation over time. Treatment with HDL (100 µg protein/ml) led to a time-dependent increase in AKT phosphorylation at Ser473 with an initial increase by 30 min, followed by a decline and then a further increase by 24 h (Figure 5A,B). In a separate experiment, we pretreated HIVCM cells with HDL for 30 min and then exposed them to OGD or control conditions (‘Normoxia’, in which oxygen and glucose levels were kept normal) for 0.5 or 4 h, and measured the effects on AKT phosphorylation (Figure 5C,D). This demonstrated that AKT phosphorylation at Ser473 was increased after the initial 30 min incubation with HDL, but then declined over the next 4 h in HDL-treated cells (consistent with the above observation), with an apparently greater decline in cells exposed to OGD. On the other hand, we saw trends towards increases (which did not reach statistical significance) in phospho-AKT levels over time in cells that were not treated with HDL, regardless of exposure to OGD or normal conditions (Figure 5C,D). Because we detected reproducibly and statistically significantly increased phospho-AKT after 30 min of preincubation of cells with HDL, we selected 30 min of HDL treatment for further study of AKT phosphorylation. When we treated HIVCMs that were transfected with control siRNA (siCntrl) or siRNA targeting human SR-B1 (siSR-B1) with HDL for 30 min, we found that, as above, HDL treatment increased the amount of AKT phosphorylation in HIVCM treated with siCntrl (1.7-fold induction in pAKT:tAKT; Figure 5E,F). Conversely, when SR-B1 was knocked down, HDL treatment did not increase AKT Ser 473 phosphorylation, suggesting that AKT is activated by HDL in an SR-B1-dependent manner (Figure 5E,F). We interpret this as suggesting that the 30 min pretreatment of cells with HDL may prime them for protection against OGD-induced cell death by stimulation of increased AKT phosphorylation.

### Inhibition of PI3K or AKT abolishes HDL-mediated protection

**Figure 6.**
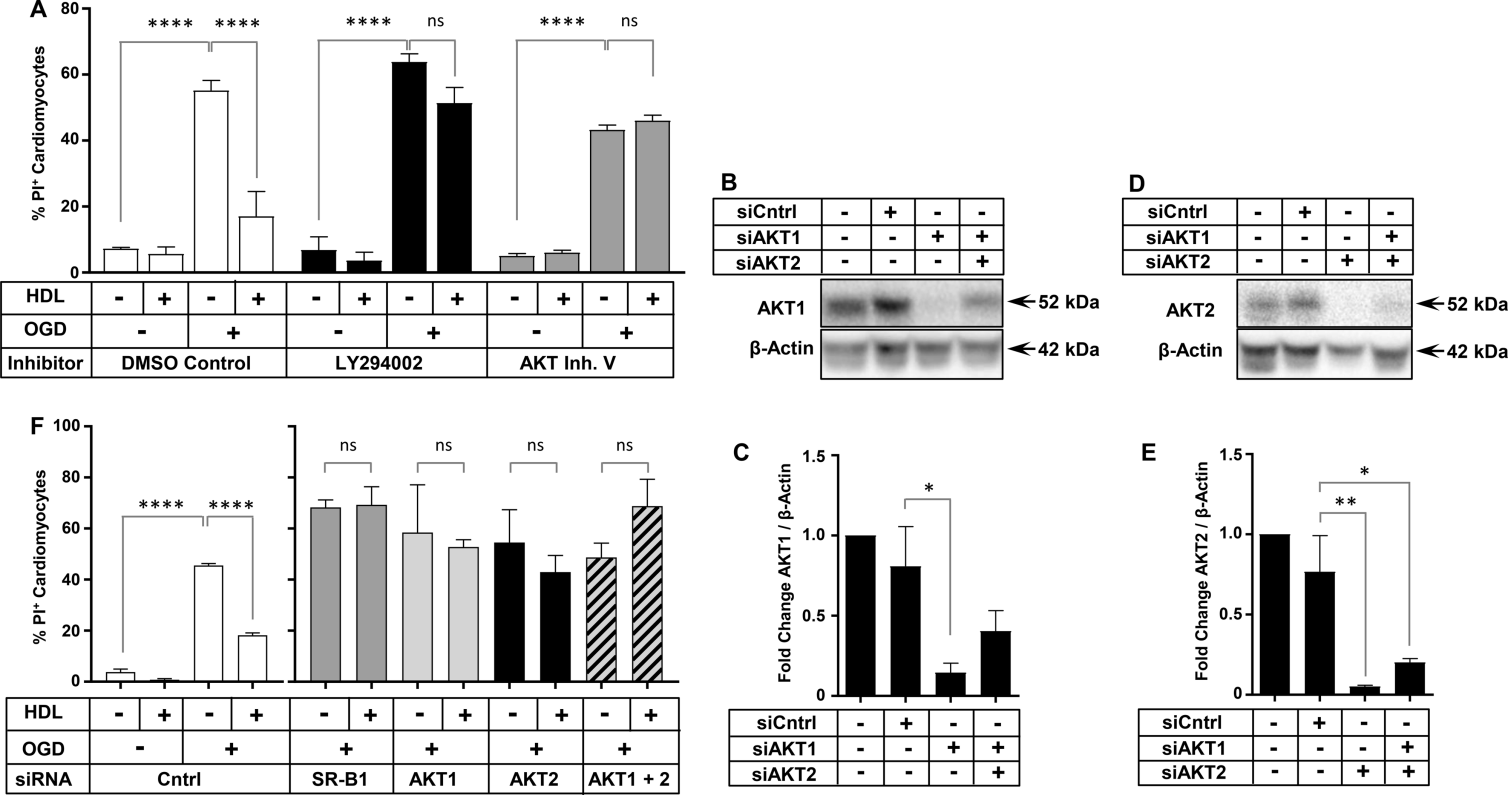
PI3K, AKT1, and AKT2 are required for HDL-mediated protection of HIVCM against OGD-induced necrosis. (**A**) Quantification of OGD-induced PI staining in HIVCM treated with or without HDL (100 μg/ml) and inhibitors of PI3K (10 µM LY294002) or AKT (3 µM AKT Inh. V) prior to 4 h of OGD. Control cells were treated with an equal volume of DMSO (0.1%). (**B**) Representative immunoblots of AKT1 and β-actin (loading control) and (**C**) quantification of fold change in AKT1/β-actin protein levels in HIVCMs transfected with the indicated siRNAs. (**D**) Representative immunoblots of AKT2 and β-actin (loading control) and (**E**) quantification of fold change in AKT2/β-actin protein levels in HIVCMs transfected with the indicated siRNAs. (**F**) Quantification of OGD-induced necrosis (% PI-positive cells) in HIVCMs transfected with the indicated siRNAs and treated with or without HDL (100 μg protein/ml) for 30 min prior to 4 h of OGD, as indicated. Data in all panels represent means ± SEM (*n* = 3 replicates). ****P < 0.0001; **P = 0.009; *P < 0.04; ns, not statistically significant (*P* > 0.33) by one-way ANOVA.

To determine the importance of the transient increase in phospho-AKT, we sought to assess whether inhibition of the activity of AKT or of PI3K (an upstream activator of AKT) impaired the ability of HDL to protect against OGD-induced necrosis. The treatment of cells with the PI3K inhibitor LY294002, or with the pan-AKT inhibitor V (AKT Inh. V), abolished HDL-mediated protection against OGD-induced necrosis in HIVCM (Figure 6A). AKT1 and AKT2 are the major isoforms of AKT expressed in the heart [15]. HIVCMs were transfected with siCntrl or siRNA targeting either AKT1 or AKT2 or a mixture of siRNAs targeting both AKT1 and AKT2. Transfection with siAKT1 or siAKT2 resulted in reduction of AKT1 or two levels by 85 and 95%, respectively (Figure 6B–E). Protection by HDL against OGD-induced necrosis was abolished when AKT1 or AKT2 alone, or the combination of AKT1 and AKT2 together were knocked down (Figure 6F), suggesting that both of the major isoforms of AKT in the heart mediate HDL-dependent protection against necrosis.

## Discussion

**Figure 7.**
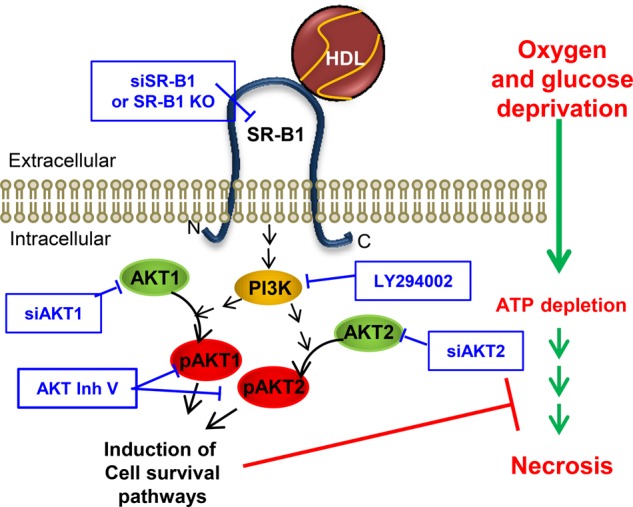
Proposed pathway for HDL-mediated protection against OGD-induced cardiomyocyte necrosis. HDL binding to SR-B1 leads to activation of AKT1 and AKT2 signaling and induction of pathways protecting cardiomyocytes from necrosis induced by OGD. Activation of signaling pathways may be indirect, as indicated by broken arrows. Pharmacological or genetic inhibition of this pathway (shown by the boxed text) prevents protection by HDL.

HDL is known to protect different cells against apoptotic cell death, induced by a variety of agents, and has been shown to exhibit cardioprotective effects both *in vivo* and *ex vivo*. We sought to determine if HDL could protect cardiomyocytes against cell death induced by OGD as a model of induced ischemia and to evaluate if SR-B1, PI3K, and AKT are involved in the protective signaling elicited by HDL. Others have shown that following 4 h of hypoxic stress, neonatal rat ventricular cardiomyocyte cell death was a result of necrosis, though reoxygenating cells for increasing lengths of time resulted in a shift of cell death from necrosis to apoptosis [24]. Similarly, we found that in HIVCM and NMCM exposed to OGD for 4 h, necrosis (detected by PI staining) was the primary driver of cell death, and that there was little to no induction of apoptosis (as evidenced by a lack of TUNEL or AxV staining), or necroptosis (as evidenced by resistance to the necroptosis inhibitor, Nec-1s). We have provided evidence that HDL protects cardiomyocytes against necrosis induced by OGD via a pathway requiring SR-B1, AKT1, and AKT2 (Figure 7). We have tested concentrations of HDL up to 100 µg protein/ml (50 µg cholesterol/ml), which corresponds to concentrations of HDL in extracellular fluid (reported to be up to 20% of plasma concentrations) [26]. Whether HDL also protects via SR-B1 against apoptosis or other forms of cell death triggered by ischemia/reperfusion remains to be determined. However, we and others have reported that HDL can protect cardiomyocytes against apoptosis induced by chemical agents, such as the cardiotoxic chemotherapeutic drug doxorubicin [27,28]. Our findings implicate a similar pathway involving SR-B1, PI3K, and AKT, suggesting that this may, indeed, be the case. This highlights the pleiotropic effects of HDL and the ability to generate protection against various forms of cardiomyocyte death.

Sphingosine 1-phosphate (S1P) is a bioactive sphingolipid that is primarily carried by HDL [29,30] and, when applied to cardiomyocytes alone, or as part of reconstituted HDL, can protect cardiomyocytes against various stresses [28,31,32]. S1P receptors (S1PRs) have been implicated in mediating S1P-dependent protection against cardiomyocyte cell death following hypoxia and rexoygenation, although different receptors have been implicated in HDL-dependent protection under different conditions; S1PR_3_ is reportedly required for HDL-mediated protection against myocardial infarction in mice *in vivo* [32], whereas mouse cardiomyocytes subjected to hypoxia/reoxygenation required both S1PR_1_ and S1PR_3_ [31], and doxorubicin-treated cultured rat cardiomyocytes required S1PR_2_ but not S1PR_1_ or S1PR_3_ [28] for HDL/S1P-mediated cytoprotection. Here, we conclude that SR-B1 is also a critical receptor for HDL-mediated protection of cardiomyocytes. It is possible that SR-B1 could function to anchor HDL in close proximity to S1PRs. Recently, it has been reported that SR-B1 and S1PR1 form complexes in the presence of S1P [33]. Alternatively, since SR-B1 is known to mediate the cellular uptake of HDL-bound lipids [34], and since HDL is known to be a major carrier of S1P in blood [29,30], SR-B1 may mediate the uptake of HDL-bound S1P, making it accessible to S1PRs. It remains to be determined if SR-B1 and S1P receptors participate with each other in HDL-mediated signaling in cardiomyocytes and by what mechanism this may occur. Regardless, our data indicate a role of SR-B1 in facilitating HDL-mediated protection against OGD-induced cardiomyocyte necrosis (Figure 7).

OGD can be applied to cardiomyocytes *in vitro* in order to mimic the *in vivo* ischemic environment and assists in defining mechanistic and direct effects of cardioprotective agents specifically on the cardiomyocyte. Necrosis is believed to be an important pathway contributing to the death of cardiomyocytes *in vivo* following myocardial infarction [35]. Other pathways of cell death also contribute. For example, apoptosis appears to be an important pathway of cardiomyocyte cell death in the infarct border zone and during reperfusion after ischemia [36]. HDL protects cardiomyocytes against apoptosis induced by the cardiotoxic agent, doxorubicin, and we have found that this protection involves SR-B1 and PI3K/AKT signaling [27]. Evidence is also emerging implicating necroptosis in cardiomyocyte cell death during infarct generation and remodeling [37]. The effect of HDL on necroptosis has not yet been examined.

Given the extensive and lethal myocardial infarctions that occur spontaneously at a young age in SR-B1/ApoE dKO mice [20], and in a high fat/high cholesterol diet-dependent manner in SR-B1^−/−^ mice either lacking LDLR or with a hypomorphic mutation in apoE [21,22], we hypothesized that increased susceptibility of cardiomyocytes lacking SR-B1 to cell death may contribute along with the occlusive coronary artery atherosclerosis that develops in these mice to the development of lethal myocardial infarction. Here, we have demonstrated that cardiomyocytes (human and mouse) depend on SR-B1 to facilitate the HDL-dependent activation of AKT and protection against cardiomyocyte necrosis triggered by deprivation of oxygen and glucose.

This research will have important implications for our understanding of how HDL protects against cardiovascular disease, beyond its roles in reverse cholesterol transport and attenuating atherosclerosis, by providing insights into the mechanisms by which it exerts direct cytoprotective effects on cardiomyocytes.

## Abbreviations

apo: apolipoprotein
AxV: annexin V
cTnT: cardiac troponin T
DAPI: 4′,6-diamidine-2′-phenylindole dihydrochloride
dKO: double knockout
DMEM: Dulbecco's modified Eagle's medium
FBS: fetal bovine serum
FITC: fluorescein isothiocyanate
HBSS: Hank's balanced salt solution
HDL: high-density lipoprotein
HIVCM: immortalized human ventricular cardiomyocytes
HRP: horseradish peroxidase
LDL: low-density lipoprotein
LDLR: LDL receptor
NMCM: neonatal mouse cardiomyocyte
OGD: oxygen and glucose deprivation
PI: propidium iodide
PI3K: phosphatidylinositol-3-kinase
siAKT1 and AKT2: siRNA against AKT1 and 2
siScram: scrambled siRNA
S1P: sphingosine-1-phosphate
S1PRs: S1P receptors
SR-B1: scavenger receptor, class B type 1
TUNEL: terminal deoxynucleotidyl transferase-mediated dUTP nick end labeling
